# Outcomes of vitrectomy for retinal detachment in a patient with Ehlers–Danlos syndrome type IV: a case report

**DOI:** 10.1186/s13256-021-02855-w

**Published:** 2021-05-20

**Authors:** Xhevat Lumi, Gaber Bergant, Anila Lumi, Mina Mahnic

**Affiliations:** 1 Eye Hospital, University Medical Centre Ljubljana, Grablovičeva ulica 46, 1000 Ljubljana, Slovenia; 2 Clinical Institute of Genomic Medicine, University Medical Centre Ljubljana, Ljubljana, Slovenia

**Keywords:** Ehlers–Danlos syndrome, Vascular type, Rhegmatogenous retinal detachment, Pars plana vitrectomy, Case report

## Abstract

**Background:**

The Ehlers–Danlos syndrome (EDS) is a group of connective tissue disorders characterized by fragile blood vessels and an increased tendency for bleeding and scarring. Here, we report the outcome of a pars plana vitrectomy for the treatment of rhegmatogenous retinal detachment in a patient with EDS type IV (vascular type).

**Case presentation:**

A 40-year-old Slovenian man with high myopia, unilateral bullous retinal detachment, and vitreous hemorrhage was referred for surgery. The patient had a history of colon perforation, muscle and arterial rupture in both lower limbs, and recurrent shoulder joint luxations. Genetic testing revealed a pathogenic mutation in the* COL3A1* gene. The patient underwent a 25-gauge three-port pars plana vitrectomy. The tendency for bleeding during surgery was prevented by endodiathermy applied to the edges of the retinal breaks. Endolaser photocoagulation was performed under air. The surgical procedure was completed with the injection of gas tamponade, followed by the patient remaining for a few days in a face-down position. Mild postoperative vitreous hemorrhage was resorbed in first week after the surgery. Postoperative extensive pigment dispersion on the posterior lens face persisted for several weeks. After the gas tamponade had resorbed, the retina was flat and remained attached during the follow-up period. Eight months after the surgery, visual acuity continued to improve from a preoperative 6/38 to 6/6.6 (Snellen chart) at the last checkup.

**Conclusion:**

A small-gauge pars plana vitrectomy with gas tamponade and laser photocoagulation under air may successfully achieve reattachment of the retina in patients with high myopia with EDS type IV and restore visual acuity.

## Background

Ehlers–Danlos syndrome (EDS) is a heritable heterogeneous group of connective tissue disorders characterized by joint hypermobility, skin hyperextensibility, and tissue fragility leading to significant bruising and atrophic scarring [1]. According to the Villefranche nosology, EDS is classified into six major groups [2].

EDS type IV is characterized by changes in the vascular structure. These patients are prone to arterial, digestive, and obstetrical complications. Congenitally thin and fragile arterial walls lead to arterial dissection and tears, bleeding, and hematomas [3]. Although this tendency is higher in large- and medium-sized arteries, all anatomical areas can be affected [3]. It is therefore assumed that surgery for rhegmatogenous retinal detachment (RRD) in patients with EDS type IV has the potential to be complicated by arterial bleeding and consecutive scarring.

Very few reports have been published on the occurrence of retinal detachment in patients with EDS [4–7], and only two of these describe a clinical case of RRD that was managed by a pars plana vitrectomy (PPV) [6, 7]. In both of the cases described, the patients underwent several surgeries due to redetachment with proliferative vitreoretinopathy, ultimately ending up with silicone oil tamponade and worse visual acuity [6, 7].

There are different types of surgical approaches for RRD, including pneumatic retinopexy, scleral buckling, and PPV [8]. The type of surgery chosen depends on the complexity of the detachment, age of the patient, and the surgeon’s preference. Primary PPV is indicated in complex retinal detachments [8]. Here, we report the case of a bullous subtotal retinal detachment and a vitreous hemorrhage managed by a small-gauge PPV in a patient with EDS type IV.

## Case presentation

A 40-year-old Slovenian man with high myopia was referred to the Eye Hospital, University Medical Centre Ljubljana, Slovenia complaining of a 2-day deterioration in the visual acuity of his right eye (OD). He described the presentation as a curtain-like dark zone in the inferior part of the visual field that progressively enlarged superiorly. He denied any eye injury.

**Fig. 1:**
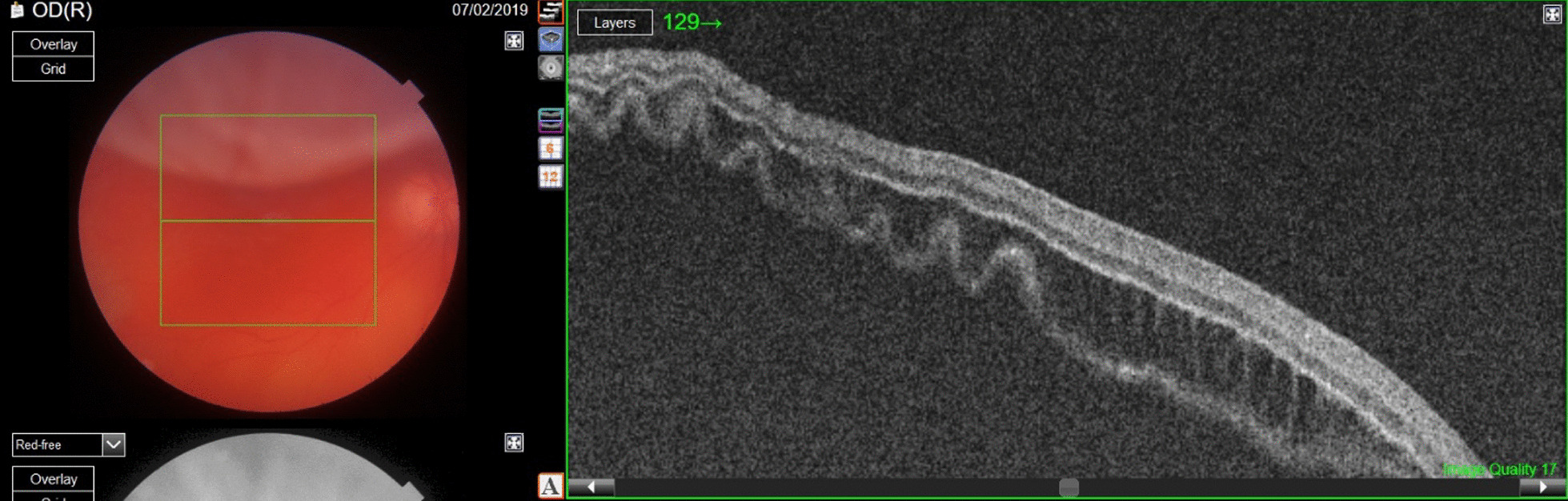
Left: Color fundus photograph of the right eye shows bullous retinal detachment with mild vitreous hemorrhage. Right: Optical coherence tomography (OCT) scan demonstrates macula-off retinal detachment

On presentation, the patient’s best corrected visual acuity (BCVA) in the right eye was 6/38 (Snellen chart) and the intraocular pressure was 10 mmHg. Slit-lamp examination of the anterior segment revealed pigment dispersion on the corneal endothelium; otherwise, the status was normal. Indirect ophthalmoscopy showed a vitreous hemorrhage and a macula-off subtotal bullous retinal detachment superiorly with two U-shaped retinal tears (Fig. 1).

The patient had high myopia and had undergone photorefractive keratectomy on both eyes 9 years previously. His previous refraction was − 9.50 Dsph (diopter sphere) in the right eye and − 5.50 Dsph in the left eye. Ophthalmoscopy of the left eye showed multiple degenerative changes on the retinal periphery.

The medical history of the patient included colon perforation, muscle and arterial rupture in both lower limbs, and recurrent shoulder joint luxation, as well as suspected EDS. His family history for connective tissue diseases was negative. The patient underwent genetic testing for EDS, which showed a pathogenic mutation in the* COL3A1* gene that is associated with EDS type IV (vascular type). The inheritance pattern of this type of EDS is autosomal dominant and there is typically a heterozygous mutation in the* COL3A1* gene that encodes type III collagen [1].

### Variant description and interpretation

Whole-exome sequencing indicated the presence of a pathogenic heterozygous missense variant in the* COL3A1* gene. Heterozygous variant c.1052G>T (LRG_3t1) in the* COL3A1* gene causes substitution of the amino acid glycine with valine at position 351 in the amino acid sequence coded by* COL3A1*. The variant was previously established as pathogenic, in line with the corresponding American College of Medical Genetics and Genomics/Association for Molecular Pathology (ACMG/AMP) criteria for interpretation of sequence variants, based on the following. First, the variant was reported to be pathogenic in a patient with vascular EDS (PS4_MOD) [9]. Second, the variant affects a glycine residue in a Gly-X-Y tripeptide repeat of the triple-helical region, which is a well-established mechanism of pathogenicity in collagenopathies (PM1) [10]. Third, the variant was not present among approximately 141,000 controls in the gnomAD project v2.1.1 (PM2) [11]. These findings indicate a missense variant in a gene for which missense variants are a common mechanism of disease. Fourth, with the gene also demonstrating evolutionary intolerance for missense variants (PP2) [11], multiple algorithms for theoretical pathogenicity predictions, including MutationTaster2 and MetaSVM, provide a unanimously pathogenic prediction of the effects of the variant on protein function, which is further supported by the location of the variant affecting an evolutionary conserved amino acid (PP3) [12, 13]. In accordance with ACMG/AMP standards and guidelines for the interpretation of sequence variants, this variant was therefore classified as a class 5–pathogenic variant [14].

**Fig. 2:**
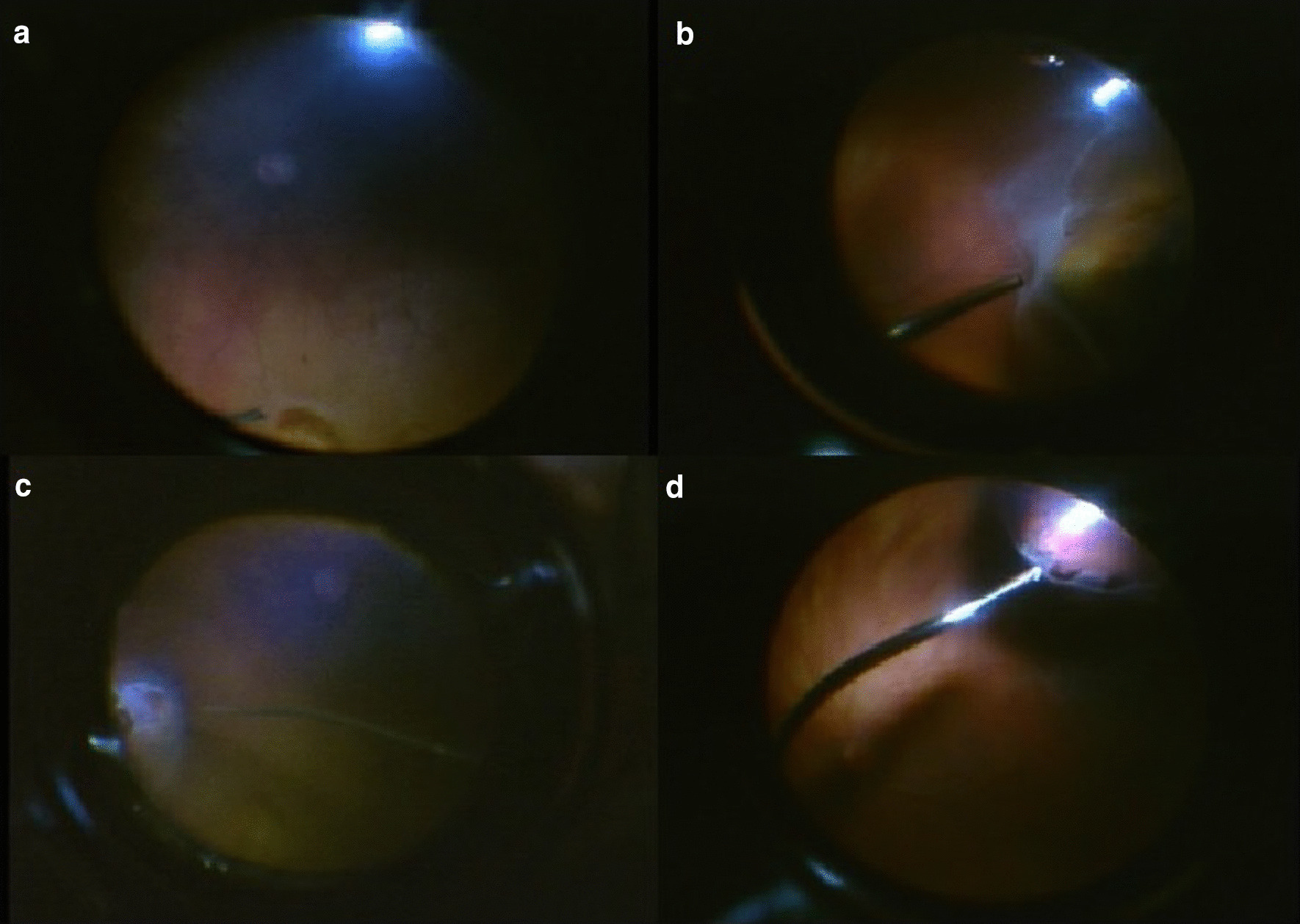
Screenshots of the intraoperative video taken during the vitrectomy of the right eye, demonstrating several retinal breaks in four quadrants at the retinal periphery. **a** Two breaks at 12 o’clock, **b** breaks at 9 o’clock, **c** breaks at 3 o’clock, **d** breaks at 7 o’clock

A 25-gauge pars plana vitrectomy was performed. After cleaning the vitreous hemorrhage, eight more retinal breaks were found positioned in all four quadrants, which could not be seen preoperatively due to the vitreous hemorrhage (Fig. 2).

**Fig. 3:**
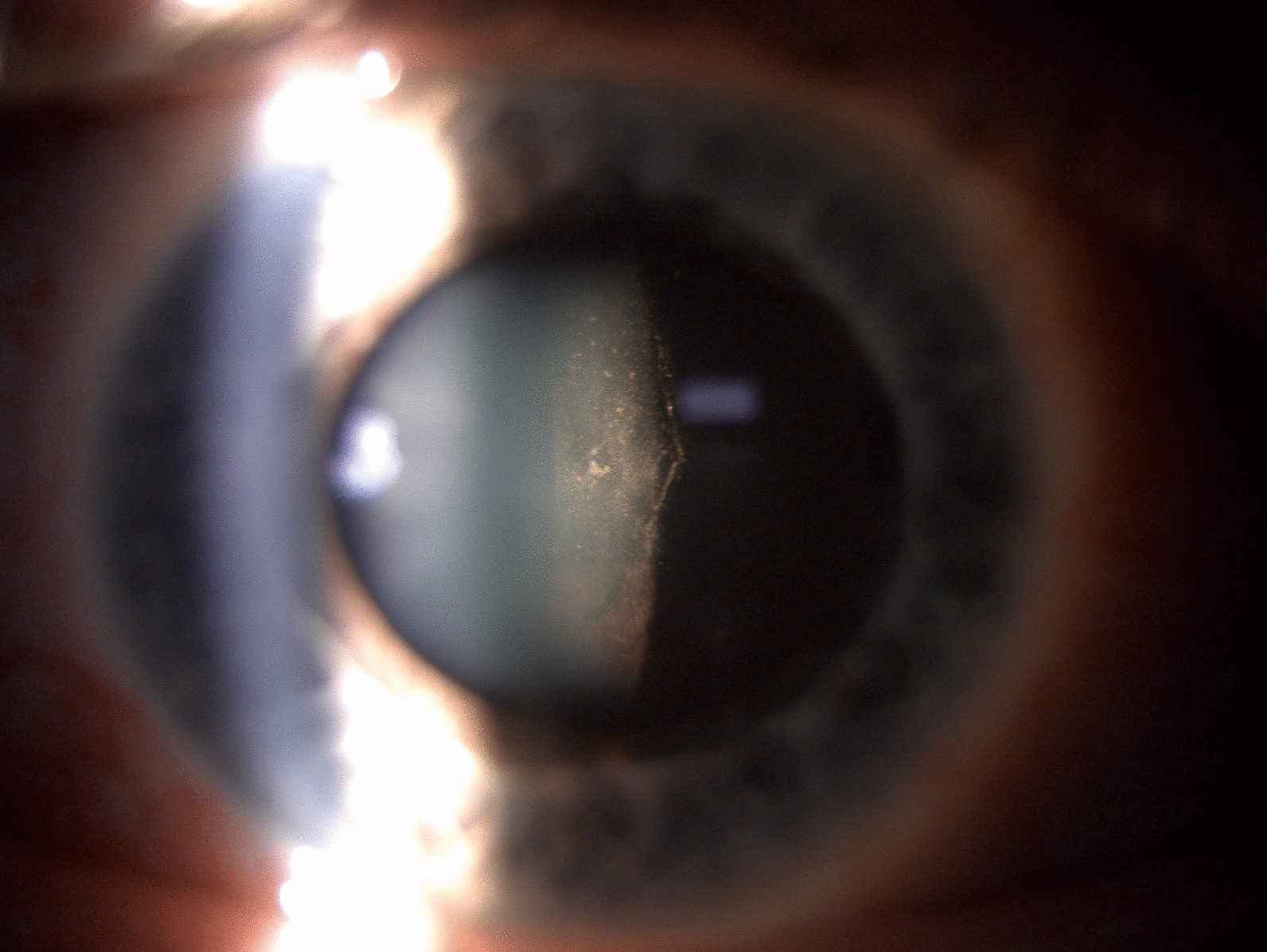
Postoperative slit lamp photograph of the anterior segment of the right eye showing extensive pigment dispersion on the posterior lens face

During the surgery, bleeding was prevented by endodiathermy applied at the edges of the retinal breaks. After shaving the vitreous periphery, fluid/air exchange was done. Laser photocoagulation around the retinal tears was performed under air. The endotamponade at the end was 10% perfluoropropane (C3F8) gas. The patient was instructed to maintain a face-down position for a few days after the surgery. Immediately after the surgery, we noted both a mild vitreous hemorrhage which resorbed within 1 week and extensive pigment dispersion on the posterior lens face which persisted for several weeks (Fig. 3). Otherwise, there were no other complications.

**Fig. 4:**
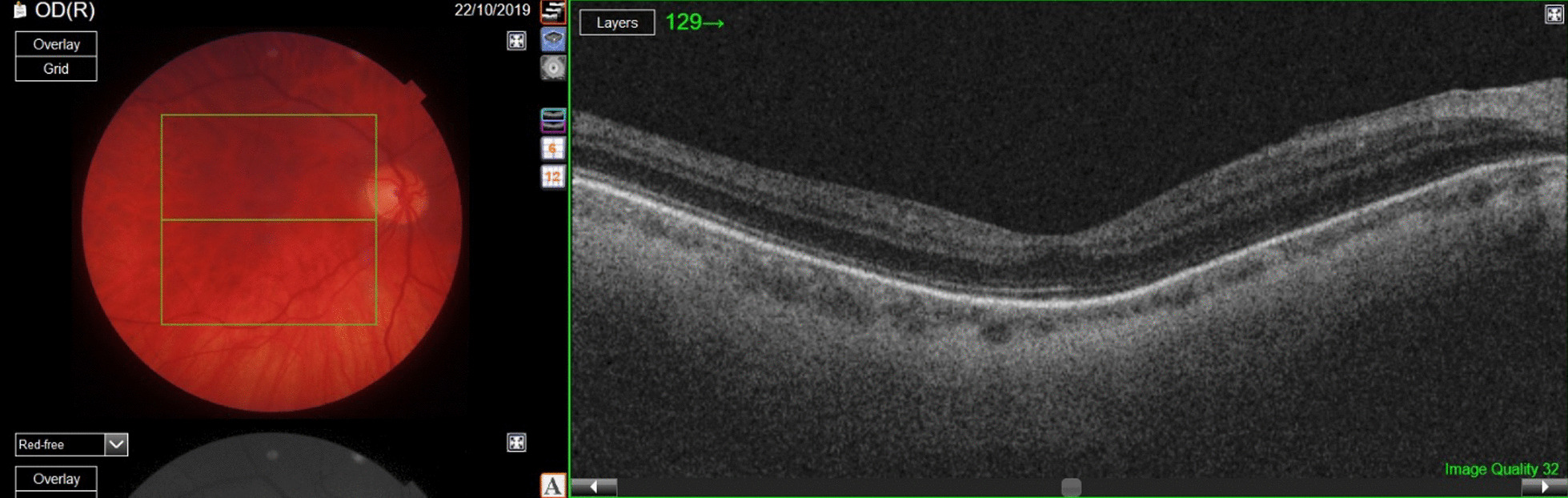
Left: postoperative color fundus photograph of the right eye showing flat retina; right: optical coherence tomography scan of the macular area demonstrating restored retinal layers

The patient underwent several postoperative examinations. At the last checkup, 8 months after the surgery, the retina remained attached. Optical coherence tomography (OCT) of the macula lutea showed normal retinal structure with distinctive layers (Fig. 4). The slit-lamp examination of the anterior segment revealed an initial posterior subcapsular cataract. Since the patient was satisfied with the functional outcome, he was not scheduled for cataract surgery. The BCVA of the right eye was 6/6.6 and the intraocular pressure was 9 mmHg.

## Discussion

The 2017 international classification of EDS recognizes 13 subtypes which are defined by major (high diagnostic specificity) ± minor (lesser diagnostic specificity) clinical criteria that are suggestive of a specific subtype [1]. Due to the vast genetic heterogeneity, phenotypic variability, and clinical overlap of the different subtypes of Ehlers–Danlos syndromes, a definitive diagnosis is reached on the basis of molecular confirmation of a causative genetic variant. Molecular diagnosis provides information on the inheritance pattern, recurrence risk, and prognosis [1].

Many ophthalmological changes have been described in EDS, such as convergence insufficiency, blue sclera, dry eye syndrome, keratoglobus, high myopia, and retinal tears [15]. Other studies have described angioid streaks, macro- and microstructural changes to the cornea, microcornea, spontaneous corneal rupture or, following minor trauma, corneal hydrops, severe conjunctivochalasis, fragility of blood vessels with recurrent vitreous hemorrhage, scleral rupture, and dislocation of the lens [16–21].

The worst affection of the eyes has been described in brittle cornea syndrome, which is characterized by very thin corneas, early keratoconus, progressive keratoglobus, blue sclera, high myopia, retinal detachment, and a higher rate of enucleation due to the changes after a globe rupture, in addition to a wide variety of extra-ocular manifestations [22].

Type IV EDS is typically caused by a mutation in the* COL3A1* gene, which encodes an important component of collagen type III. Collagen type III is found in tissues of the walls of blood vessels, intestinal walls, lungs, and skin. Since corneal collagen fibrils are composed of collagen types I and V and the vitreous body collagen fibrils are types II, IX, V/XI, and VI collagens, the main affected structure in EDS type IV can be sclera, which is composed of collagen I and III [23–25].

Apart from several reports on structural changes, there are very few publiations on retinal detachment and its management in EDS. Bodanowitz et al. reported a case of a patient with RRD in EDS type VI managed by PPV [6]. These authors described several complications during the surgery, such as choroidal detachment and intensive bleeding. The closure of the sclerotomies was also difficult due to the very thin sclera. Because of redetachment with proliferative vitreoretinopathy, the patient needed two revitrectomies. The final visual acuity after the last surgical procedure with silicon oil tamponade was 6/60 [6].

A similar course of the disease was reported by Whitlow et al. [7]. Their patient with EDS type VIa and bullous retinal detachment required several surgeries with scleral patch grafts and 20-gauge PPVs because of retinal detachment with proliferative vitreoretinopathy. The patient ended up with a worse anatomical and functional outcome, namely, a visual acuity 1/60 [7].

Our patient with EDS type IV presented with an acute worsening of visual acuity in a high myopic eye that has already undergone corneal refractive surgery. The detachment was bullous in superior quadrants accompanied by a vitreous hemorrhage. We decided to perform a small gauge PPV (25-gauge) with the aim of reducing intraoperative trauma. Intraoperatively, we found a total of ten retinal breaks localized in all four quadrants. Since this type of EDS has a higher tendency for bleeding, we prevented intraoperative bleeding by coagulation to the edges of the retinal breaks by endodiathermy. By performing laser photocoagulation under air, the energy needed for photocoagulation was lower since the retina is closely attached to the retinal pigment epithelium, which reduces cell dispersion and the possible occurrence of scarification or postoperative proliferative vitreoretinopathy.

Eight months after the surgery the retina remained attached. An OCT scan of the macula did not show structural changes. Apart from incipient lens opacification, there were no further changes during the follow-up period. The patient was satisfied with the functional result of the surgery and was not scheduled further for cataract surgery.

In this single case of EDS type IV, we were able to reattach the retina and restore visual function.

## Conclusions

Small-gauge vitrectomy can reduce intraoperative trauma and avoid intensive bleeding. Performing endodiathermy to the edges of the retinal breaks and laser photocoagulation under air reduces the tendency for bleeding and cell dispersion. Therefore, we conclude that small-gauge PPV can produce good outcomes in high myopia retinal detachment in patients with EDS type IV.

## Data Availability

The data that support the findings of this study are available from the medical records. The authors confirm that all data underlying this case are fully available without restriction and can be accessed by contacting Xhevat Lumi (xhlumi@hotmail.com).

## Abbreviations

EDS: Ehlers–Danlos syndrome
BCVA: Best corrected visual acuity
OCT: Optical coherence tomography
PPV: Pars plana vitrectomy
RRD: Rhegmatogenous retinal detachment
